# Production, Biosynthesis, and Commercial Applications of Fatty Acids From Oleaginous Fungi

**DOI:** 10.3389/fnut.2022.873657

**Published:** 2022-05-19

**Authors:** Xin-Yue Zhang, Bing Li, Bei-Chen Huang, Feng-Biao Wang, Yue-Qi Zhang, Shao-Geng Zhao, Min Li, Hai-Ying Wang, Xin-Jun Yu, Xiao-Yan Liu, Jing Jiang, Zhi-Peng Wang

**Affiliations:** ^1^School of Marine Science and Engineering, Qingdao Agricultural University, Qingdao, China; ^2^Key Laboratory of Sustainable Development of Polar Fishery, Ministry of Agriculture and Rural Affairs, Yellow Sea Fisheries Research Institute, Chinese Academy of Fishery Sciences, Qingdao, China; ^3^Key Laboratory of Bioorganic Synthesis of Zhejiang Province, College of Biotechnology and Bioengineering, Zhejiang University of Technology, Hangzhou, China; ^4^Jiangsu Key Laboratory for Biomass-Based Energy and Enzyme Technology, Huaiyin Normal University, Huaian, China; ^5^School of Environmental Science and Engineering, Suzhou University of Science and Technology, Suzhou, China

**Keywords:** oleaginous fungi, triacylglycerols, regulation strategy, fatty acids, Commercial application

## Abstract

Oleaginous fungi (including fungus-like protists) are attractive in lipid production due to their short growth cycle, large biomass and high yield of lipids. Some typical oleaginous fungi including *Galactomyces geotrichum, Thraustochytrids, Mortierella isabellina*, and *Mucor circinelloides*, have been well studied for the ability to accumulate fatty acids with commercial application. Here, we review recent progress toward fermentation, extraction, of fungal fatty acids. To reduce cost of the fatty acids, fatty acid productions from raw materials were also summarized. Then, the synthesis mechanism of fatty acids was introduced. We also review recent studies of the metabolic engineering strategies have been developed as efficient tools in oleaginous fungi to overcome the biochemical limit and to improve production efficiency of the special fatty acids. It also can be predictable that metabolic engineering can further enhance biosynthesis of fatty acids and change the storage mode of fatty acids.

## Introduction

**Figure 1:**
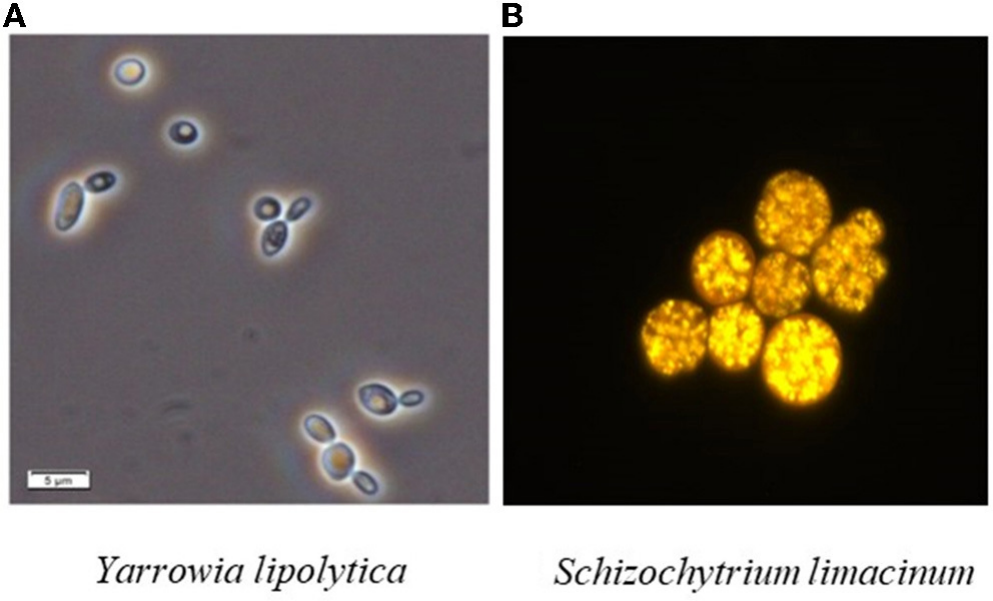
Lipid bodies from different oleaginous fungi. **(A)** Wang et al. (4); **(B)** Fillet et al. (5).

Similar to vegetable lipids, microbial lipids mainly include neutral fatty acids (FAs), free FAs and phospholipids (1, 2). Moreover, they share the same existence form with animal and plant lipids, i.e., existing in the cell structure such as membrane with constant content or forming lipid droplets or fat particles in the cytoplasm (3). The bright spheres in Figure 1 are lipid droplets in cells of different types of strains. Specifically, the outer layer of lipid droplets is a monolayer composed of phospholipids and specific proteins, and the inner core is mainly neutral lipids such as triacylglycerol (TAG) and sterol ester (SE) (4–7).

Microbial lipids are also widely used in the production of biodiesel. Excessive consumption and environmental damage caused by fossil fuels have hindered economic sustainable development (8, 9). Therefore, finding renewable and clean energy that can replace fossil energy is an important prerequisite for the development of green economy, energy conservation, emission reduction, and environmental protection (2). Methyl or ethyl fatty acid ester is obtained from methyl or ethyl esterification of FAs. The cellular lipids are mainly produced in the form of free FAs and acylglycerols (mostly as triglycerides) and are stored in the globular organelles called lipid bodies. Transesterification of microbial lipids is an essential step in microbial lipid production at both laboratory and commercial scale. Direct transesterification can considerably reduce costs, increase sample throughput and improve lipid yields (in particular fatty acid methyl esters, FAMEs). Fatty acid ethyl esters (FAEEs) are typically produced via the chemical transesterification of plant lipids and animal fats. Biosynthesis of FAEEs is limited by the supply of precursor lipids and acetyl-CoA (10–13).

Oleaginous fungi, as lipid-producing microorganisms, are attractive in lipid production due to their short growth cycle, large amount of biomass and high yield of lipids (14, 15). Some filamentous fungi species have been reported able to accumulate polyunsaturated fatty acids (PUFAs), such as *Mortierella isabellina, Mucor circinelloides, Pythium ultimum* (2, 9). PUFAs play a vital role in human body; (PUFAs) belonging to the ω-3 and ω-6 classes are also substantial as precursors of eicosanoids or being structural components of various membrane phospholipids (9, 16, 17). Table 1 summarizes some high-yield fungi species and their lipid content.

**Table 1:** Lipid contents of some fungi.

**Species**	**Carbon source**	**Lipid content (%)**	**PUFAs (%)**	**References**
*Aurantiochytrium* sp.	Glucose	72.4	+25 (DHA)	(18)
*Umbelopsis vinacea*	Glucose	63.55	+	(19)
*Aurantiochytrium* SW1	Fructose	44	52.3 (DHA)	(14)
*Aurantiochytrium* sp. T66	Glycerol	55	40 (DHA)	(20)
*Mortierella alpina*	Potato industry wastes	40	35 (ARA)	(21)
*Aurantiochytrium* sp. SY25	Glucose	/	59.98	(22)
*Mucor wosnessenskii* CCF 2606	Soybean	6.7 ± 0.3	8.5 ± 0.2 (GLA)	(15)
*Mortierella isabelline* ATHUM 2935	Glucose (commercial)	83.3	/	(23)
*Thraustochytrium* sp. T18	Glucose	46.9	35.2 (DHA)	(24)
*Galactomyces geotrichum* TS61	molasses	69.6	23.67 (LA)	(25)
*Mortierella alpina*	Glycerol (crude)	33.3	49.2 (LA)	(26)
*Mortierella alpina* CCFM698	Glucose	31.5	26.7 (EPA)	(27)

As fungus-like protists, *Thraustochytrids* are progressively studied for his or her quicker growth rates and high lipid content (28). *Thraustochytrids* were first reported in 1936 and have attracted much attention since 1990 due to their high yield of FAs (29). The accumulated lipids account for more than 50% of dry cell weight (DCW), of which more than 25% is docosahexaenoic acid (DHA) with extremely high economic value. *Aurantiochytrium* sp. is a kind of *thraustochytrids*, which has a high yield of PUFAs, especially DHA (9). At present, the research on FA synthesis from *Aurantiochytrium* sp. is mainly focused on the optimization of culture conditions to increase the yield of unsaturated FAs, especially DHA (7).

Different from oleaginous yeasts, accumulation of fatty acids with important functions is the most attractive point of oleaginous fungi. However, unlike oleaginous yeasts, the recent developments of production, biosynthesis, and commercial applications of fatty acids from oleaginous fungi, have not been reviewed. In this article, we tried to summarize the studies of fatty acids from oleaginous fungi, and provided a point of reference.

## Regulation of Fungal Fatty Acid Fermentation

Fungal lipid fermentation can be divided into two stages, i.e., the cell proliferation and the lipid accumulation (30). During the cell proliferation stage, cells proliferate and metabolize vigorously, with the nutrients in the medium consumed rapidly. During the fatty acid accumulation stage, the nitrogen source is exhausted but the carbon source is sufficient in the medium, which makes cells stop proliferating for the most part, with the lipid synthesis becoming the dominated metabolic activity (31–33).

According to the characteristics of fungal lipid fermentation production, controlling the nutrient composition of medium and regulating environmental conditions are a common strategy to promote lipid biosynthesis in lipid fermentation engineering. At present, three regulation strategies are widely adopted for lipid fermentation. The carbon-to-nitrogen (C/N) ratio, carbon and nitrogen sources, pH, incubation temperature and dissolved oxygen are the main factors influencing fatty acid production (18, 34). Nevertheless, other factors also play a crucial role in microbial activity, such as minerals (e.g., sulfur, zinc and phosphorus) and vitamins (e.g., thiamine and biotin) (35). Moreover, secondary metabolites (like citrate) are also an influencing factor for lipid production (34).

### Promoting Lipid Accumulation by Nutrients Restriction

As for the de novo lipid accumulation, concentrations of nitrogen and carbon sources respectively determine the biomass content and the quantity of lipids in general (36). Accordingly, the C/N ratio is significant for the accumulated lipid content and the oleaginous microbial biomass (36–39). Previous studies demonstrate that the lipid accumulation is boosted at a C/N molar ratio of greater than 20. It is worth noting that the lipid production declines instead at the C/N ratio higher than 70 in some cases (40). Therefore, to achieve a high-level lipid accumulation, the initial C/N molar ratio should be optimized (41–43). For the lipid fermentation of the *Thraustochytridae* sp. PKU#Sw8, the increase in DHA production coincided with the up-regulation of gene expression under nitrogen-deficient culture conditions (44). Chen et al. (45) optimized the culture of *Thraustochytrid* sp. PKU#SW8 under optimal culture conditions (glycerol, 20 g/L; peptone, 2.5 g/L; 80% seawater; pH 4.0; 28°C), the cell mass, DHA concentration and yield of PKU#SW8 were increased to 7.5 ± 0.05 g/L, 2.14 ± 0.03 g/L and 282.9 ± 3.0 mg/g, respectively, on a 5-L scale fermentation.

Cellular lipid content and lipid yield were 62.2% and 0.205 g/g glucose, respectively, using a medium with a carbon to nitrogen (C/N) molar ratio of 6.1 and a C/P molar ratio of 9,552 (46), which means that the accumulation of lipid can also be regulated by limiting phosphorus in the medium. As a consequence, the regulation of phosphorus and sulfur limitation is of great significance for the production of lipids from nitrogen-rich crude materials (41, 45, 47, 48).

### Promoting Lipid Accumulation by Small Molecules

Some small molecules can also regulate the accumulation of lipids (37, 48, 49). Li et al. (50) cultured *Thraustochytriidae* sp., a kind of marine oleaginous protists, by addition of different levels of sodium nitrate (1-50 mM) or urea (1-50 mM) in fermentation culture has a significant effect on fatty acid synthesis. They found that urine (50 mM) culture the cells accumulated 1.16 times the ω-3 PUFAs, of which DHA accounted for 49.49% and docosapentaenoic acid (DPA) was 5.28% compared with the original culture conditions. To sum up, it is easy to control lipid accumulation by small molecules, which is of great significance for the optimization of lipid production conditions (48, 51).

The accumulation of biomass and lipid-synthesizing fungi in any oily substance is highly affected by factors such as pH, temperature, light, and ventilation. Temperature change is also one of the factors affecting lipid accumulation (52, 53). They found that the low temperature has a significant impact on the formation of DHA, which can increase the DHA content from 43 to 65% of the total fatty acids. Low temperature may increase DHA content by facilitating a relatively large amount of substrates to enter the polyketide synthase (PKS) pathway (52).

### Promoting Lipid Accumulation by Using Different Fermentation Modes

Generally speaking, there are three ways of microbial fermentation: batch culture, fed-batch culture and continuous culture (38, 54–57). The batch culture is the most widely used for lipid fermentation. Wang et al. (58) studied *Schizochytrium* sp. PKU#Mn4 and *Thraustochytrid* sp. PKU#Mn16, found that the largest DHA yields were 21% and 18.9%, and the yields were 27.6 mg/L-h and 31.9 mg/L-h, respectively in in 5-L bioreactor fermentation operated with optimal conditions and dual oxygen control strategy. The production of DHA increased by 3.4 times and 2.8 times (g/L) respectively. *Rhizopus* sp. using solid-state fermentation and submerged fermentation can produce valuable alternative feed ingredient due to their high protein and the well-balanced lipid content and amino acid profile (59).

In addition, electro-fermentation is a promising technology that can improve the performance of biological processes. When lipids are produced yeast *R. toruloides* under electro-fermentation conditions, the proportion of saturated FAs increases significantly from 37 to 50% (60).

## Extraction of Fungal Fatty Acid

The conventional methods of wall breaking mainly include the following: grinding method, acid treatment, cell autolysis method, repeated freezing and thawing, ultrasonication and enzyme treatment (61–65). Among them, the autolysis method has simple steps and low cost, but has poor crushing result and low lipid yield; the enzymatic treatment method has mild conditions and no damage to intracellular substances, but is expensive and cannot be used for large-scale treatment. Ultrasonication is one in every of the additional normally used strategies. Using ultrasound to reinforce the synthesis of designer lipids, researchers have discovered an eco-friendly technique for enhancing the synthesis of designer lipids with numerous nutritional values (66).

The extraction of lipids is mostly done with low-boiling organic solvents. Commonly used solvents are ether, petroleum ether and chloroform. At present, the commonly used extraction methods of microbial lipids are as follows: acid heat method, Soxhlet extraction method, and supercritical CO_2_ extraction method (67–71). Among them, the Soxhlet extraction method is relatively accurate, but it is time-consuming and consumes too much organic solvent; the acid-heat method, although the yield is low, is fast and simple, and is suitable for the operation of multiple samples; the supercritical CO_2_ extraction method has high instrument requirements and requires Strictly control parameters (67, 71). The process of extracting lipid from fungi using acid-catalyzed predicament, microwave, and rapid ultrasonic-microwave treatment can create it have a high extraction rate, up to 70% (w/w) content (71). It is a novel green extraction method (63).

### Cost Estimation of Fungal Fatty Acids

Take DHA as an example for cost estimation (72). If all the carbon sources needed to produce DHA were glucose, the amount of glucose required to produce 1 ton of biomass would be 2.78 tons. At the 2021 glucose price of us $903.9 per ton, it would cost US $2,512.84 to produce one ton of biomass. If there is only 50% lipid content in 1 ton of biomass, 40% of the lipid content is DHA (73). That's 0.2 tons of DHA. If one ton of DHA is produced, the calculated cost of glucose is $12,564.2. All this takes into account only glucose substrates, but if you add in other cost factors, including water and electricity, publicity, equipment, and so on, the cost of DHA increases further (72).

Recovery processes downstream of the fermenter typically contribute 60–80% to the cost of production of a fermentation product, therefore the fermentation step contributes only around 20–40% to the total production cost. The above analysis leads to the conclusion that the *Schizochytrium limacinum* grown on glucose cannot provide DHA cheaper than fish oil at present. If the biomass was used simply as an aquaculture feed additive, the downstream processing requirements would mostly disappear, although on the basis of equal DHA content, the biomass would still be more expensive than fish oil. Hence the need for cheaper nutrients for growing thraustochytrids (72).

In the future development, it is very important to improve production efficiency and reduce cost. First of all, in terms of carbon sources, the focus should be on replacing glucose while maintaining high biomass and lipid yields. In addition, lignocellulose hydrolysate may prove to be an inexpensive source of carbon for biomass production, which can efficiently metabolize xylose, and xylose metabolic engineering may help reduce fermentation costs (74). Metabolic engineering is also very important (75, 76). Therefore, strains should be improved, complete metabolic flux analysis should be carried out, and the protein engineering field should be evaluated with the goal of metabolic engineering, etc., in order to maximize the fatty acid production in the biomass.

Taking into consideration from another angle, developing new valuable products such as enzymes, and cell wall polysaccharides, during fungal fermentation besides fatty acids, would effectively reduce the cost. This “fungal-based biorefinery” strategy has not been applied in the studies, it may be the another choice to make the production of fatty acids more feasible (77).

## Fatty Acid Production From Raw Materials

Glucose is the most basic carbon source of microorganisms. Many studies explore the glucose-based lipid accumulation of fungi, and the lipid content can reach higher than 70% (w/w) (41, 78–80). However, the large-scale production of microbial fatty acids with glucose as a raw material will face the problems of “competing with people for food” and “competing with food for land”, which necessitates the search for other suitable raw materials to reduce the costs (9, 81, 82). Recently, some cheap and available “raw materials” have been widely concerned, such as lignocellulose, non-grain sugar raw materials and commercial wastes (9, 79, 83). Table 2 summarizes some high-yielding fungal species that use non-glucose as substrates for lipid accumulation.

**Table 2:** Non-glucose substrates for lipid production.

**Species**	**Carbon source**	**Lipid content (%)**	**References**
*M. circinelloides*	Xylose	17.2-17.7	(84)
*Ashbya gossypii*	Xylose	55	(85)
*M. circinelloides* Q531	Mulberry branches	28.8 ± 2.85	(86)
*M. circinelloides* ZSKP	Kitchen vegetable waste	21.4	(87)
*M. alpina* CBS 528.72	Potato waste	40	(21)
*Aurantiochytrium* sp. YLH70	Jerusalem artichoke	46.9% (DHA)	(88)
*Aurantiochytrium* sp. T66	Glycerol	55	(65)
*Aspergillus caespitosus* ASEF14	Sago processing wastewater (SWW)	37.2	(89)
*Cutaneotrichosporon curvatus*	Lignocellulose	63	(90)

### Lignocellulose

Lignocellulose is constituted by hemicellulose, cellulose and lignin (91). In recent years, lignocellulosic biomass has been recognized as a potential alternative feedstock to produce biofuels (9). The fatty acid production with lignocellulosic biomass includes the following two steps: (1) the degradation of biomass to corresponding monosaccharides by heat-acid treatment or enzyme hydrolysis, and (2) the biodegradable sugar fermentation by promising oleaginous microorganisms (92–95). Lignocellulose cannot be directly utilized, but must be hydrolyzed, which produces compounds that inhibit the growth of fungi, such as furan aldehydes, weak acids, and aromatic compounds, during the pretreatment process (96). The cumulative deleterious effects of some inhibitors (such as furfural, formic acid, acetic acid, and vanillin) on fatty acid accumulation in oleaginous fungi have been investigated (96, 97). Intasit et al. (98) used an integrated biotechnology, fungi and yeast to bioconvert lignocellulosic biomass into biodiesel, first pretreatment of the fungus, the fungus *Aspergillus tubingensis* TSIP 9 lipid yield 121.4 ± 2.7 mg/g-EFB (empty fruit bunch), the integrated biotechnology can greatly facilitate the conversion of lignocellulosic biomass to biodiesel feedstock is a cost-effective and sustainable biotransformation. Zhang et al. (84) deeply analyzed the effects of corn stover hydrolyzate on lipid accumulation by using xylose metabolism engineering strains of *M. circinelloides* strains. The results showed that the fatty acid contents of the engineered *M. circinelloides* strains were increased by 19.8% (in Mc-XI) and 22.3% (in Mc-XK), respectively, compared with the control strain.

Glucose and xylose coexist in lignocellulose hydrolysate. Lipid-producing yeasts consume glucose first and then xylose, and even some lipid-producing yeasts are unable to utilize xylose. Therefore, lignocellulose hydrolysate suffers from long fermentation cycle and low substrate utilization.

### High-Carbonhydrate Plant Materials

Jerusalem artichoke is a kind of perennial plant resistant to barren, cold and drought. The planting of Jerusalem artichoke should not occupy cultivated land and other agricultural lands (99). The storage form of sugar in Jerusalem artichoke is inulin, which is a polyfructose linked by β-2,1 glycosidic bond with a glucose residue at the end (100). Yeast or molds can accumulate large amounts of lipids from inulin hydrolysates (101–103). In the medium containing inulin, fatty acids can be produced and the lipid content and biomass of cells can be changed. By converting the inulinase gene, the gene accumulates higher fatty acids (100). *Aurantiochytrium* sp. YLH70 can produce lipid in a medium with 695 mL/L hydrolysate of Jerusalem artichoke. The biomass higher biomass (32.71 g/L) and DHA content (46.9% of the total fatty acid) (88).

### Commercial Organic Wastes

The combination of low-cost organic compounds contained in agro-commercial waste and the cultivation of lipid-producing microorganisms can effectively achieve the effect of accumulating lipids (104, 105). Lipid-producing microorganisms use some forms of carbon sources and nutrients for growth and fatty acid accumulation. Organic waste usually contains organic particles, which may be an ideal and inexpensive substrate for microbial fatty acid production, but the chemical composition of organic waste affects the lipid production of different species (104, 106). Lipid-producing yeasts can also transform commercial organic wastes into lipid (105, 107). Crude glycerol is a by-product of biodiesel industry, which is usually treated as commercial waste (64). The engineered strain of the filamentous fungus *Ashbya gossypii* can produce microbial lipids, whose efficiency is improved by three genomic manipulation methods. Using organic commercial waste as a raw material, the strain can accumulate lipid at about 40% of DCW (85). The use of commercial waste to produce lipid is also of great significance to environmental governance.

## Synthesis Mechanism of Fatty Acids in Fungi

Essentially, the synthesis of microbial lipids is similar to that of animal and plant lipids. After the carboxylation of acetyl-CoA, saturated or unsaturated FAs are generated through chain extension and desaturation, and then triacylglycerols (TAGs) are formed.

### General FA Biosynthesis

**Figure 2:**
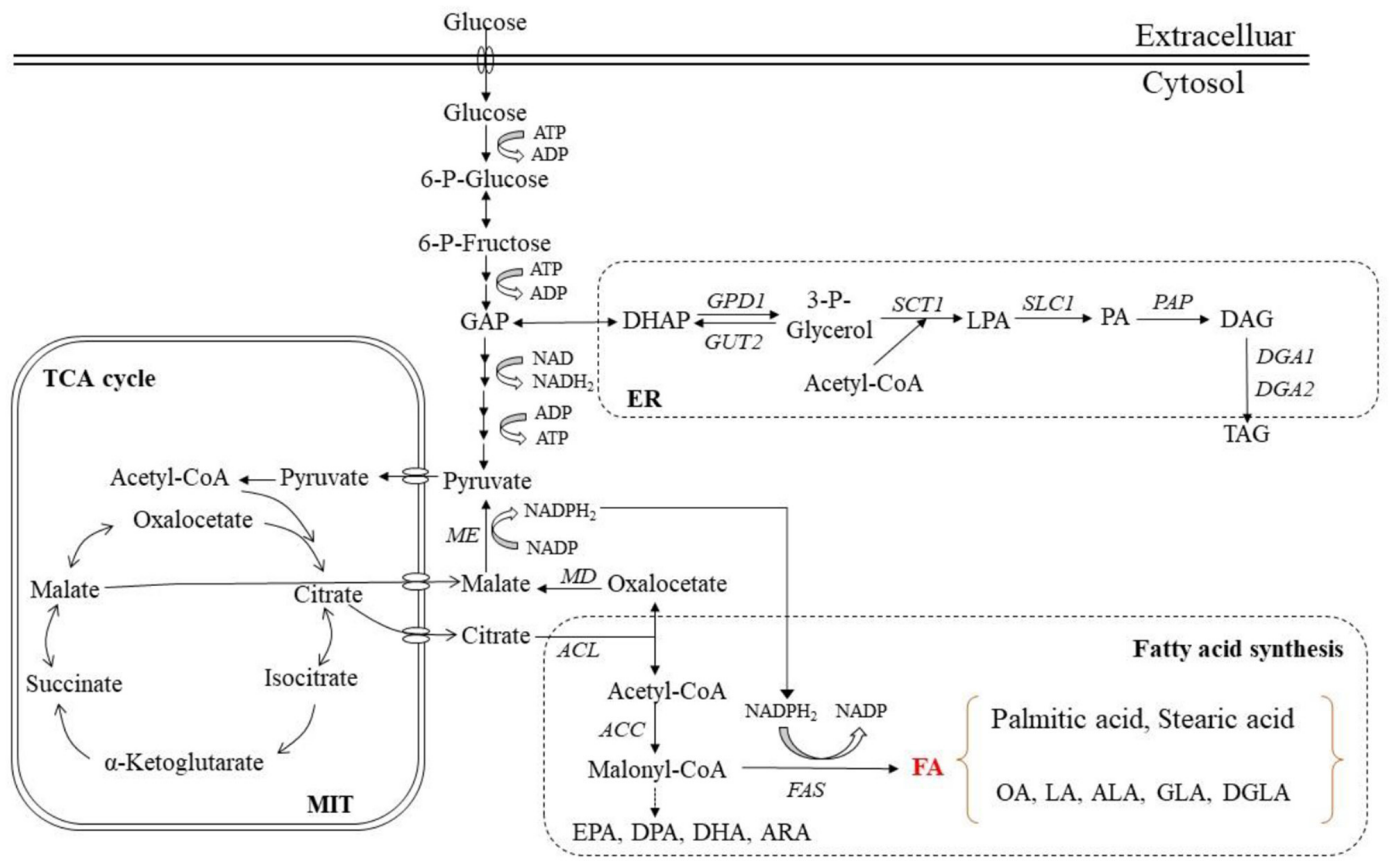
TAGs and fatty acid synthesis in microbial cells. MIT, Mitochondria; ER, Endoplasmic reticulum; ACL, ATP-citrate lyase; FAS, Fatty acid synthase; MD, Malic dehydrogenase; ME, Malic enzyme; ACC, Acetyl-CoA carboxylase; TCA, Tricarboxylic acid cycle; DAG, Diacylglycerol; PA, Phosphatidic acid; TAG, Triacylglycerol; FA, Fatty acid; OA, Oleic acid; LA, Linoleic acid; ALA, α-Linolenic acid; GLA, γ-Linolenic acid; DGLA, Dohomo-γ-linolenic acid; ARA, Arachidonic acid; EPA, Eicosapentaenoic acid; DPA, Docosapentaenoic acid; DHA, Docosahexaenoic acid.

The synthesis of FAs in microbial cells requires acetyl-CoA acting as the precursor of FAs and a sufficient supply of NADPH to provide reducibility for the synthesis. It is generally believed that when nitrogen is depleted, the activity of AMP-deaminase increases. This can supplement ${\text{NH}}_{4}^{+}$ for various metabolisms, decrease intracellular AMP level and the activity of isocitrate dehydrogenase (IDH) activated and cause the accumulation of isocitrate in mitochondria (108). Aconitase (AT) in mitochondria is able to catalyze the conversion of over-accumulated isocitrate to citric acid. The citric acid is then transported to the cytoplasm, where the ATP-citrate lyase (ACL) helps its cracking catalysis to acetyl-CoA and oxaloacetic acid (OAA). As a result, abundant acetyl-CoA is produced as the precursor of FAs (109). Acetyl-CoA directly participates in the FA synthesis, while oxaloacetate is first reduced to malate dehydrogenase (MD) and then undergoes oxidative decarboxylation in the presence of malic enzyme (ME) to release NADPH (110). Studies have shown that ME can regulate lipid accumulation in oleaginous microorganisms (111). Accordingly, if the activity of ME is inhibited, the lipid accumulation will decrease. This is because although many reactions in the cellular metabolic network can produce NADPH, the NADPH required by FA synthesis comes almost entirely from ME-catalyzed reactions (111). Catalyzed by acetyl-CoA carboxylase (ACC), acetyl-CoA and CO_2_ were transformed into monoyl-CoA. Multiple reactions can be continued in the presence of FAS. Acetyl-CoA combines with ACP to form acetyl-ACP, and malonyl-CoA and acetyl-CoA yield acyl-CoA via a condensation reaction. The three steps of reduction, dehydration and re-reduction are continued, and the FA chain extends by two carbon atoms. NADPH is taken as the reducing cofactor by FAS, and two NADPH molecules are required in each step of the acyl-CoA chain elongation. The chain is repeatedly extended to the desired length of the synthetic organism, and then some FAs are desaturated to form unsaturated FAs (112–114). The related reactions and enzymes are shown in Figure 2.

### PKS Pathway

The synthesis of some special FAs in microorganisms may be related to PKS. PKS is a sophisticated molecular machine responsible for synthesizing polyketides, which are natural products from the secondary metabolism with similarities to FA (115–117).

**Figure 3:**
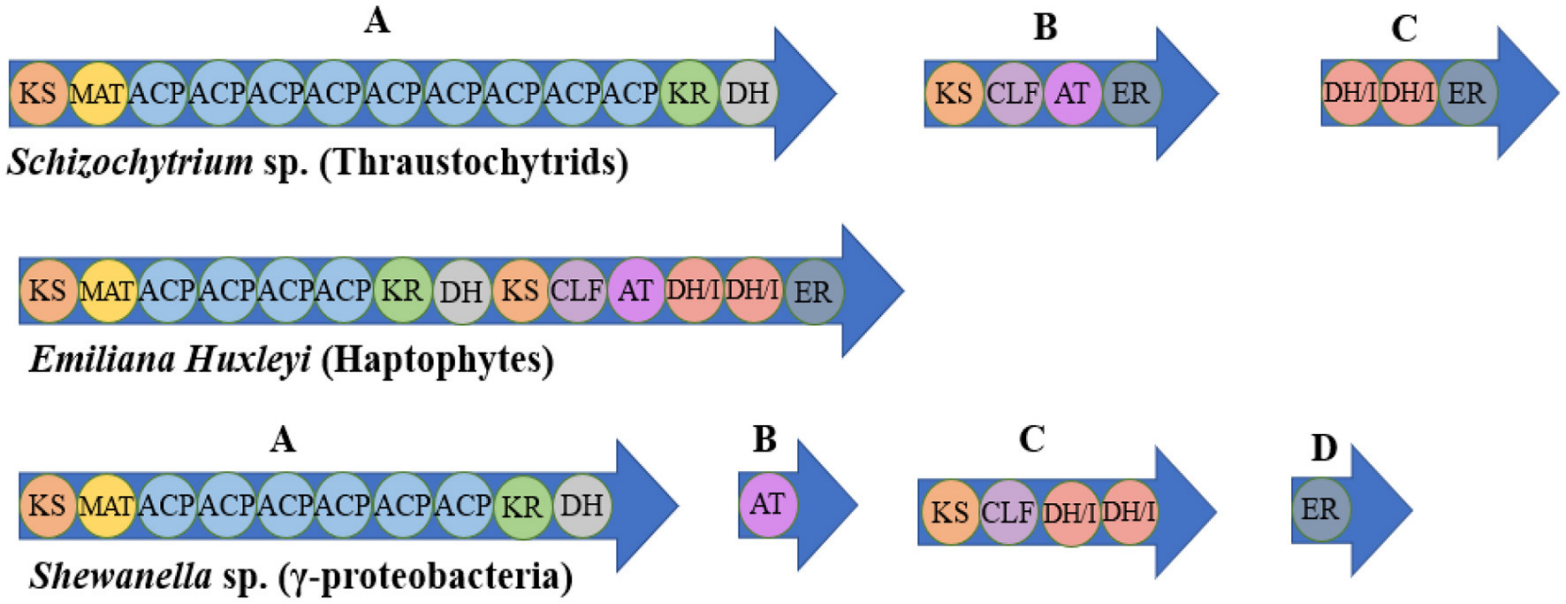
Examples of PUFA synthase organization in various representative organisms. KS, β-ketoacyl synthase; MAT, malonyl-CoA: ACP transacylase; ACP, acyl-carrier protein; KR, β-ketoreductase; DH, dehydratase; CLF, chain length factor; AT, acyl transferase; ER, enoyl-reductase; DH/I, dehydratase/isomerase.

The DHA synthesis in *Thraustochytrium, Schizochytrium limacinum, Aurantiochytrium* sp. is considered to involve the PKS pathway. The successive condensation reactions of precursors catalyzed by PKS can form a variety of polyketides, and then numerous complex compounds are generated through modification reactions such as methylation, redox, glycosylation and hydroxylation (116, 118). In terms of structure and properties, PKS can be divided into three types: modular type (type I), repetition type (type II) and chalcone type (type III). The PKS found in fungi is mostly type I, which is large multifunctional proteins encoded by a single gene. It has multiple similar modules, and some domains are reused in the compound synthesis (117). The type I has a multidomain architecture whose active sites were distributed on large modules, while the type II is composed of monofunctional enzymes, with catalytic sites separated on different proteins (119). Type III polyketide synthases (PKSs) produce secondary metabolites with diverse biological activities, including antimicrobials (120, 121). In contrast to types I and II, type III PKSs are dimers of ketone synthases that undergo a series of reactions such as initiation of primer substrates, decarboxylation condensation of extended substrates, ring closure of growing polyketide chains and aromatization, and produce a variety of biologically active aromatic compounds (122). The PKS pathway of some species is shown in Figure 3 (119).

### TAG Synthesis

At present, TAGs are viewed as an important form of carbon source and energy storage unit in microorganisms. The TAG synthesis pathway is triggered at the point when carbon is abundant but nitrogen is depleted in the medium. The FA biosynthesis in cytosol involves several reactions which convert the precursor, acetyl-CoA, into long-chain FAs (123). The synthesized acyl-CoA has a typical chain length of 18 or 16 carbon atoms. These C18:0 and C16:0 molecules are then delivered to the endoplasmic reticulum (ER) to be further elongated and desaturated (124–126). The TAG synthesis requires a variety of enzymes, and phosphatidic acid (PA) and diglyceride are two key intermediates in anabolic metabolism.

Generally, the TAG synthesis involves the Kennedy pathway, with glycerol-3-phosphate (G3P) and acyl-CoA serving as the direct substrates in the process (126, 127). The first step of TAG assembly is the conversion of G3P into lysophosphatidic acid (LPA) with G3P acyltransferase (*SCT1*) as the catalyst (128). Subsequently, the LPA acylation occurs to generate PA in the presence of LPA acyltransferase (*SLC1*). Further, under the action of phosphatidic acid phosphatase (PAP), PA is dephosphorylated to produce diacylglycerol (DAG) (129). Finally, TAG is formed after the DAG acylation at the sn-3 position by an acyl-CoA-independent or acyl-CoA-dependent reaction (130, 131). Regarding the acyl-CoA-independent reaction, glycerophospholipid is the acyl group donor and phospholipid DAG acyl-transferase (*LRO1*) catalyzes the process. With respect to the acyl-CoA-dependent reaction, acyl-CoA acts as the final donor of acyl group and DAG acyltransferases, i.e., *DGA1* or *DGA2*, are responsible for the catalysis. Furthermore, acting as the acyl-transferase of an acyl-CoA-dependent reaction, the steryl ester synthetase, which is encoded by *ARE1*, is proved able to promote the DAG acylation (123). The related enzymes and specific reactions are displayed in Figure 2.

### TAG Degradation

The TAGs accumulated in cells store energy for them. Once carbon becomes insufficient, TAGs would be degraded with acetyl-CoA release so that the cellular metabolism can be maintained. Initially, free fatty acids (FFAs) can be produced from TAGs in the presence of intracellular lipases (TGL3 and TGL4) (132). These FFAs are activated by FAA1 to generate acyl-CoAs, which are then transported by specific transporters (Pxa1 and Pxa2) into the peroxisome (133). Alternatively, the transportation of the produced FFAs into the peroxisome takes place first, followed by the activation to acyl-CoAs therein by acyl/aryl-CoA ligase (AAL) (134). Afterward, acyl-CoAs are degraded in the peroxisome via the β-oxidation pathway to generate acetyl-CoA.

## Metabolic Engineering of Oleaginous Fungi

Researchers have modified a variety of lipid-producing fungi to improve production efficiency of fatty acids. On the whole, these modifications can be divided into four categories: (1) enhancement of FA synthesis pathway, (2) enhancement of TAG synthesis pathway, (3) overexpression of key enzymes for providing cofactors, and (4) the blocking competitive pathway (32, 135, 136). Table 3 summarizes some studies on genetic modification of genes related to lipid synthesis.

**Table 3:** Researches about lipid synthesis by overexpressing genes or knocking-out genes.

**Genes**	**Species**	**Lipid content (%)**	**References**
*sodit*	*M. lusitanicus*	+24.6	(111)
*mt*	*M. lusitanicus*	+33.8	(111)
*ΔSnf-β*	*M. circinelloides*	+32	(137)
*g6pdh1*	*M. circinelloides*	+23-38	(138)
*g6pdh2*	*M. circinelloides*	+41-47	(138)
*leuB*	*M. circinelloides*	+67-73	(138)
*CT*	*M. circinelloides*	+51% efflux rate of [14C] citrate	(139)
*ΔCT*	*M. circinelloides*	−18% efflux rate of [14C] citrate	(139)
*Δ-15D, MFE1, PEX10*	*Y. lipolytica*	77.8	(140)
*DGA1, MFE1, PEX10*	*Y. lipolytica*	71	(141)
*ACC1*	*M. rouxii*	40	(142)
*MA-GAPDH1*	*M. alpina*	+13	(143)
*YlGSY1*	*Y. lipolytica*	+60% TAG	(144)
*IDH*	*M. alpina*	+8.2	(145)
*ER*	*S. limacinum* SR21	+47.63	(146)
Overexpression of ACL and ACC	*Schizochytrium* sp. ATCC 20888	73	(147)
*ELO3*	*Schizochytrium* sp. S31	+1.39 times DHA	(148)
*sodit-a* or/and *sodit-b*	*M. circinelloides*	+10-40	(149)

### Enhancement of FA Synthesis Pathway

Previous studies have shown that the expression of acetyl-CoA carboxylase (encoded by *accA, accB, accC* and *accD*) and thioesterase I (encoded by *tesA*) in *Escherichia coli* can speed up the FA synthesis by six times. This suggests that the catalytic reaction of acetyl-CoA carbohydrase is a rate-limiting step for FA synthesis (150–153). Overexpression of both heterologous Δ-15 desaturase (Δ-*15D*) sourced from flax and endogenous genes (*SCD, ACC1, DGA1* and Δ-*12D*) along with the deletion of endogenous *MFE1* and *PEX10* can yield a superior lipid producer (140). It is able to produce lipid with content of 77.8% and titer of 50 g/L using glucose as the substrate in a 5 L stirred-tank bioreactor (140).

Han et al. (147) overexpressed in *Schizochytrium* sp. ATCC 20888 using the strong constitutive promoter ccg1, *Schizochytrium* ATP-citrate lyase (ACL) and acetyl-CoA carboxylase (ACC). The lipid content of overexpressed strains obtained by fermentation culture can reach a maximum of 73.0%, an increase of 38.3%. However, the *ACC1* gene from mold *M. rouxii* is expressed in the *Hansenula polymorpha*, and the fat content is only 40% higher than the original (142), possibly because the fungus has a more powerful metabolic regulation system.

### Enhancement of TAG Synthesis Pathway

Diacylglycerol acyltransferase (DGAT) catalyzes the conversion of DAG and acetyl-CoA to TAG, which is the last step in the TAG synthesis (32, 154, 155). When grown in batch conditions and minimal medium, the resulting strain consumes 12 g/L cellulose and accumulates 14% (DCW) lipids (156). Blazeck et al. (141) synergistically regulated multiple key genes related to the degradation and biosynthesis of lipids in *Y. lipolytica* with a combinatorial strategy, including *MFE1, AMPD, PEX10, MAE, DGA1, ACL1, ACL2* and *DGA2*, with 57 distinct genotypes generated. The double deletion of *MFE1* and *PEX10* and the overexpression of *DGA1* were most effective for modification. After the optimization of bioreaction conditions, the engineered strain had a lipid content of 71% (DCW) and a lipid titer of 25 g/L. Markedly, a 60-fold improvement was realized over the original strain.

### Overexpression of Key Enzymes for Providing Cofactors

IDH and ME probably play a crucial role in the accumulation of lipids. When glucose-6-phosphate dehydrogenase (G6PD), 6-phosphogluconate dehydrogenase (PGD), IDH and ME are overexpressed in *M. alpina*, total FAs can be increased by 1.7 times; while ME2 is more effective in desaturation, and the content of arachidonic acid (AA) is increased by 1.5 times compared to the control (157). Glyceraldehyde 3-phosphate dehydrogenase (GAPDH) is an enzyme highly conserved in the glycolytic pathway. The lipid-producing filamentous fungus *M. alpina* was used to characterize two copies of the gene encoding GAPDH, and the overexpression strain MA-GAPDH1 increases the lipid content by about 13% (143). First, the total lipid accumulation was increased by overexpressing a malic enzyme from *Crypthecodinium cohnii* to elevate NADPH supply. Then, the inhibition effect on acetyl-CoA carboxylase was relieved by overexpressing a codon-optimized *ELO3* gene from *M. alpina*. After the above two-step engineering, contents of DHA was increased by 1.39-fold, reaching a level of 26.70% of dry cell weight, respectively (148).

The PKS cluster genes are supposed to synthesize PUFAs in *S. limacinum*. Ling et al. (146) improved lipid production domain expression by homologous recombination knocking out two enolate reductase (ER) genes located on the PKS cluster. The addition of triclosan as a modulator of the ER domain resulted in a 51.74% increase in PUFA production and a 47.63% increase in lipid production.

### Blocking Competitive Pathways

The main competitive pathways blocking the lipid accumulation in microbial cells include β-oxidation of FAs, synthesis of phospholipids and conversion of phospho-enol-pyruvic acid (PEP) to oxalacetic acid. The β-oxidation occurs in peroxisomes, and peroxisome biogenesis is generally downregulated by the deletion of *PEX3, PEX10* and *PEX11* so that the degradation of TAGs can be prevented in commercial strains (158, 159). As an important intermediate metabolite, malate, its subcellular location and concentration have significant effects on fungal lipid metabolism. Yang et al. (149) deleted the two plasma membrane malate transporters “2-oxoglutarate:malate antiporter” (named SoDIT-a and SoDIT-b) of *M. circinelloides* WJ11 and analyzed their effects on growth ability, lipid accumulation and metabolism. Their results showed that the lipid content of the mutant was increased by ~10–40% compared to the control strain, indicating that defects in plasma membrane malate transport lead to an increase in malate for lipid synthesis.

Additionally, the genes involved in the β-oxidation pathway are often the deletion targets to increase lipid accumulation (160). After the characterization and deletion of the *YlGSY1* gene encoding glycogen synthase, Bhutada et al. (144) increased the TAG accumulation of the engineered strain by 60% as compared with the wild-type strains. This proves that glycogen synthesis is a competing pathway, and its elimination is beneficial for the production of neutral lipids.

## Commercial Applications of Fungal FAs

Recently, many researchers make efforts to explore the applications of microbial lipids in various fields from the food and health industry to the production of plasticizers, lubricants, spices and pesticides. Additionally, they are also promising intermediates in fine chemicals and other industries. This part mainly introduces the applications of polyunsaturated fatty acids from fungi. Polyunsaturated fatty acids (PUFAs) have received increasing attention for their beneficial effects on human health. PUFAs refer to long-chain FAs containing two or more double bonds, mainly including linoleic acid (LA), conjugated linoleic acid (CLA), γ-linolenic acid (GLA), AA, eicosapentaenoic acid (EPA), DHA, which are mostly the precursors of bioactive substances. They have the capabilities of anti-aging, anti-oxidation and anti-inflammation and are able to inhibit the formation, proliferation and metastasis of tumor cells and treat heart disease, hypertension, etc. Table 4 summarizes the production of unsaturated fatty acids by some fungi and their application functions.

**Table 4:** Sources and uses of various unsaturated fatty acids.

**Category**	**Species**	**Functions**
DHA	*Thraustochytrium, Schizochytrium limacinum, Aurantiochytrium* sp.	Conducive to retinal development, promote brain development, prevent cardiovascular disease
EPA	*Diasporangium* sp, *Mucor, Mortierella alpine Cunninghamell*	Lowers cholesterol levels, resists arteriosclerosis, prevents Alzheimer's disease and vision loss, improves brain function, is added to healthy food and baby food
ARA	*Mortierella, Mortierella alpina, Mortierella isabellina*	Promoting brain and nervous system development
ALA	*Saccharomyces cerevisiae*	Inhibiting thrombotic diseases, reducing blood pressure and blood lipids
GLA	*Mucor hiemalis, Mucor circinelloides, Rhizopus, Zygomycetes*	Plays an important physiological role in cardiovascular, immune, reproductive and endocrine systems, lowers blood sugar and blood lipids
Palmitic acid	*Schizochytrium*	Treatment of inflammation in cells and organs caused by excessive consumption
LA	*Galactomyces geotrichum, Mortierella alpina, Mucor circinelloides*	Reducing blood lipid, soften blood vessels, reducing blood pressure, promoting microcirculation
DGLA	*Mortierella alpina, Pythium, Entomophyhora*	Treating atherosclerosis

## DHA

DHA is an important member of the ω-3 PUFA family and has a wide distribution. Among marine organisms such as fish, shrimps, crabs, and seaweeds, DHA is particularly abundant in lipids of deep-sea fish (161). It can be produced in the *Thraustochytrium, Schizochytrium limacinum, Aurantiochytrium* sp. and so on. Although DHA can be produced from α-linolenic acid, the reaction rate is low, which thus necessitates its intake from diet (7). It accumulates in retinal tissue and gray matter in general and plays a key role in early visual and neural development (162). Besides, it is conducive to the development of the retinal, neuronal and immune systems at embryonic and post-natal stages (163, 164) and is effective to prevent cardiovascular disease, maintain brain and learning functions and protect inflammation response systems in adulthood (165). As a nutrient, DHA can be used in maternal and infant products. DHA and other unsaturated FAs in microalgae can be fully digested and absorbed by some aquatic organisms to meet the growth and development of juvenile fish and improve their survival rate (166).

## EPA

EPA belongs to the ω-3 series of PUFAs. Natural phospholipids containing EPA are mainly found in the eggs and muscle tissues of marine animals. EPA can be produced in Mucorales, *M. alpina* and so on. Studies have revealed that EPA is able to protect the heart against the deleterious effects of sepsis in female rats. The following two reasons account for this beneficial action: (1) The anti-inflammatory activity of EPA which reduces the oxidative stress and preserves the energy metabolism through an increase in UCP3; (2) the incorporation of EPA in membrane phospholipids that increases the vasodilator reserve of the coronary microvessels (167, 168). EPA and DHA have various physiological functions such as reducing cholesterol content, resisting arteriosclerosis, preventing Alzheimer's disease and vision loss and improving brain function (164, 169). EPA and DHA are usually added to health foods and baby foods. High levels of EPA and DHA can be used as drugs to treat cardiovascular and cerebrovascular diseases, e.g., hyperlipidemia and arteriosclerosis. The preparation of high-purity EPA and DHA is the current deep processing target of fish lipids (27, 170). EPA is also an important functional component of breast milk, which is essential for the development of the baby's brain and vision. Therefore, more and more researchers are committed to applying EPA to infant milk powder in hope of improving its nutritional value through simulating the nutrients in breast milk (171).

## ARA

ARA is an important member of the ω-6 PUFA family and has a wide distribution. Like DHA, which plays an important role in the development of infants' brains and retinas, it is one of the important factors affecting the quality of infant milk powder (21, 172). *Mortierella*, a fungus of the order *Mucor*, is a good producer of ARA. Research shows that ARA and DHA together constitute 20% of the weight of the human brainstem and are mainly concentrated in the outer neuron membrane and iliac sheath (173, 174). Bieren et al. (175) discovered that ARA metabolites can promote the occurrence of acute inflammation and produced pro-inflammatory mediators, such as PGE2 and PGfc. Lipoxin A_4_ derived from ARA can promote the degradation of lipid mediators. ARA accounts for 15–17% of the total FAs in skeletal muscle, which benefits the growth and repair of skeletal muscle tissue (176). Studies have shown that ARA supplementation can stimulate prostaglandin release and induce skeletal muscle hypertrophy through COX-2 dependent pathways (177).

## GLA

GLA, one of the essential FAs, is an important component of biofilm (15). The microbial sources of GLA are mainly fungi and microalgae. For example, the microbial sources of GLA mainly include *Spirulina* (*S. maxima, S. arthrospirulina*), *Mucor* (*M. rucus* and *M. microflora*), *Rhizopus*, and *Crucifera* (15, 178, 179). GLA plays a significant physiological role in cardiovascular, immune, reproductive and endocrine systems. It is important because of its nutritional value and medicinal applications (180). GLA can act on lipoprotein enzyme and lipase to affect the formation and expression of TAGs, total cholesterol and very-low-density and low-density lipoproteins, thus having the capacity to lower blood lipid (181).

### High-Value Chemicals Production From Oleaginous Fungi

In addition to the above-mentioned polyunsaturated fatty acids, some of these strains can also produce high levels of squalene and carotenoids, two other compounds of commercial value with rapidly growing market potential (182). Squalene has antioxidant and anticancer activities with broad applications in food and cosmetics industries. Besides, squalene has been used as vaccine adjuvant in vaccine formulations (43, 183). Since, the demand for squalene has increased during the last decade, microbial production of squalene has been investigated as a promising alternative source for traditional extraction methods from shark liver or plant lipids (184). Microbial strains are capable of producing non-polluting, low-cost, high-quality and sustainable sources of squalene, which is the main direction of the lipid-based biofuel industry.

*Aurantiochytrium* strains have the potential to produce large amounts of squalene, and *Aurantiochytrium* is known for its potential to produce large amounts of polyunsaturated DHA on a large scale (185). Furthermore, *Thraustochytrid* reported the co-production of squalene and DHA from inexpensive feedstocks such as organic solvent pretreatment spruce hydrolysis (186).

## Conclusion

Accumulation of fatty acids with important functions is the most attractive point of oleaginous fungi. However, the cost limits application of the functional fatty acids. It is very important to improve production efficiency and reduce cost of the fatty acids. Replacing glucose with raw materials, while maintaining high biomass and lipid yields, was considered a feasible strategy. More fundamentally, many metabolic engineering strategies have been developed as efficient tools in oleaginous fungi to overcome the biochemical limit and to improve production efficiency of fatty acids. Particularly, the special kind of functional fatty acid can be enhanced by modifying the biosynthetic pathway with much higher yield. It also can be predictable that metabolic engineering can change the storage mode of fatty acids, even simplify the extraction. Thus, oleaginous fungi can be developed as hosts for high-value fatty acids and fatty acid-derived chemicals.

## Author Contributions

Z-PW and X-JY made important contributions to the study conception and design. X-YZ participated in the drafting and revision of the manuscript. BL and B-CH conducted data analysis and interpretation. F-BW, Y-QZ, S-GZ, and ML performed literature research and organization. X-YL, JJ, and H-YW revised the manuscript. All authors read and approved this manuscript and agreed to be responsible for all aspects of the research to ensure the data accuracy and integrity of this work.

## Funding

This research was supported by the Natural Science Foundation of Shandong Province (ZR2020MC003), the First Class Fishery Discipline Programme in Shandong Province, a special talent programme One Thing One Decision (YishiYiyi) Programme in Shandong Province, China, the Zhejiang Provincial Natural Science Foundation of China (No. LY18C010004), and the Talent Research Foundation of Qingdao Agricultural University (663/1117023, 663/1120036, and 663/1120058).

## Conflict of Interest

PW was employed by company Linyang Group. The remaining authors declare that the research was conducted in the absence of any commercial or financial relationships that could be construed as a potential conflict of interest.

## Publisher's Note

All claims expressed in this article are solely those of the authors and do not necessarily represent those of their affiliated organizations, or those of the publisher, the editors and the reviewers. Any product that may be evaluated in this article, or claim that may be made by its manufacturer, is not guaranteed or endorsed by the publisher.
